# From “Eating for Two” to Food Insecurity: Understanding Weight Gain Perspective During Pregnancy Among Malaysian Women

**DOI:** 10.3390/healthcare13101099

**Published:** 2025-05-08

**Authors:** Shahrir Nurul-Farehah, Abdul Jalil Rohana, Noor Aman Hamid, Zaiton Daud, Siti Harirotul Hamrok Asis

**Affiliations:** 1 Environmental Health Research Centre, Institute for Medical Research, National Institutes of Health, Ministry of Health Malaysia, Shah Alam 40170, Selangor, Malaysia; 2 Department of Community Medicine, School of Medical Sciences, Universiti Sains Malaysia, Kubang Kerian 16150, Kelantan, Malaysia; na.hamid@usm.my; 3 Nutrition Division, Ministry of Health Malaysia, Putrajaya 62000, Malaysia; 4 Family Health Unit, Selangor State Health Department, Shah Alam 40100, Selangor, Malaysia; ctharirotul@yahoo.com.my

**Keywords:** gestational weight gain, pre-pregnancy BMI, maternal obesity, diabetes, household income, maternal food insecurity, mixed-methods design, Malaysia

## Abstract

Background/Objectives: Gestational weight gain (GWG) is a critical determinant of pregnancy outcomes; however, studies on factors contributing to suboptimal GWG in developing countries, including Malaysia, remain limited. Methods: This study employed an explanatory sequential mixed-methods design, with the quantitative phase conducted between January and March 2020, followed by the qualitative phase from July 2020 to March 2021 in Selangor. The qualitative phase aimed to explain the factors influencing suboptimal (inadequate and excessive) GWG identified in the quantitative phase. Inclusion criteria included Malaysian women aged 18 and above who had suboptimal GWG (either inadequate or excessive) from the quantitative phase. Exclusion criteria included women who refused participation. Of the 475 participants from the quantitative phase, 20 with suboptimal GWG were purposively selected for in-depth telephone interviews using a semi-structured interview protocol. Data were analysed using thematic analysis. Results: Three key themes emerged: (1) the impact of pre-pregnancy overweight and obesity, shaped by unhealthy lifestyles, social influences, and limited access to nutritious food and physical activity; (2) the management of diabetes during pregnancy, contributing to inadequate GWG due to psychological responses, restrictive behaviours, and barriers to dietary guidance; and (3) financial constraints in middle- and low-income households, leading to income vulnerability, financial crises, and food insecurity. Conclusions: This finding highlights the urgent need for targeted interventions to optimize GWG, emphasizing pre-pregnancy health optimization, enhanced diabetes management, and strategies to mitigate financial constraints and food insecurity among pregnant women.

## 1. Introduction

Nutrition plays a vital role in ensuring the health and well-being of both the mother and foetus during pregnancy. An adequate and balanced dietary intake during this critical period supports optimal foetal development and reduces the risk of pregnancy-related complications. Nutrition in pregnancy has become a priority on international public health agendas, particularly following the adoption of the Sustainable Development Goals (SDGs) and the United Nations’ declaration of the Decade of Action on Nutrition (2016–2025). Gestational weight gain (GWG), as part of pregnancy nutrition, is considered one of the indicators of the effectiveness of national health services and programs.

Gestational weight gain (GWG) is defined as the amount of weight gained between the time of conception and the onset of labor and consists of both the product of conception and maternal physiological changes [1]. The revised 2009 Institute of Medicine (IOM) recommendations recommend a weight gain range based on maternal pre-pregnancy BMI, with a more restrictive range for obese pregnant women to mitigate adverse pregnancy outcomes. The revised guidelines reflect changes in pregnant women’s demographics over the previous decade, with more women being overweight or obese at conception [1]. Suboptimal gestational weight gain refers to inadequate GWG or excessive GWG [2]. A meta-analysis of 1,309,136 pregnancies indicated that less than half of pregnant women in the US, Europe, and Asia gained the recommended amount of weight [3]. Excessive GWG has been linked to poor maternal and foetal outcomes, including increased risk of hypertensive disorders in pregnancy, gestational diabetes mellitus (GDM), foetal macrosomia, prematurity, delivery complications, postpartum weight retention, and subsequent maternal and offspring obesity and cardiometabolic diseases [4,5]. Meanwhile, inadequate GWG is associated with anemia in pregnancy, preterm births, and low birth weight [6]. Both inadequate and excessive GWG have a higher rate of maternal and perinatal death and severe morbidity [7]. GWG is influenced by multiple factors, including age, parity, ethnicity, education level, income, pre-pregnancy BMI, smoking status, diet, physical activity, and comorbidities [8,9]. Additionally, socio-cultural beliefs, social networks, and structural disparities influence the behaviours and perceptions of women with suboptimal GWG [10].

Although adequate GWG is important for favourable pregnancy outcomes, there is a paucity of data on the prevalence and modifiable risk factors of GWG, particularly in developing countries such as Malaysia. Most published studies focus on developed countries with distinct sociocultural contexts than developing countries, despite the fact that overnutrition is increasing while undernutrition persists in these countries [11,12]. A 2016 nationwide survey in Malaysia found that 14.6% of pregnant mothers were obese [13], and the prevalence was higher in hospital-based studies [14]. Moreover, most of the studies on GWG focused on the association between GWG and foetal and maternal outcomes, while existing local studies were limited to quantitative assessments of GWG predictors [15,16,17], and only one study examined the influence of socio-cultural norms and the environment on dietary nutrient intake and physical activity during pregnancy [18]. Farhana and colleagues found that inadequate GWG was prevalent among pregnant women in Kelantan, Malaysia, with low education, advanced maternal age, and pre-pregnancy overweight and obesity significantly associated with inadequate GWG [16]. However, the mechanism underlying the findings was unable to be determined due to the nature of the study. Additionally, nutrition during pregnancy is a critical determinant of maternal and foetal health, yet pregnant women are often influenced by conflicting narratives—including the cultural norm of “eating for two” and the reality of food insecurity, which limits access to nutritionally adequate food. These opposing forces can contribute to both excessive and inadequate GWG, complicating efforts to promote optimal maternal nutrition. Additionally, while suboptimal GWG is associated with poor maternal and foetal outcomes, food insecurity further compounds these risks and has been associated with adverse pregnancy outcomes, including maternal anemia, gestational diabetes mellitus (GDM), pre-eclampsia, preterm birth, intrauterine growth restriction (IUGR), and low birth weight. It is crucial to recognize that pregnancy represents a stage in a continuum of a woman’s health, where lifestyle choices made before, during, and after pregnancy collectively influence maternal and feotal outcomes. Thus, healthy living should extend beyond pregnancy and encompass the entire reproductive lifespan.

Therefore, this present study addresses these gaps by identifying GWG predictors and explaining underlying mechanisms that result in suboptimal GWG across multiple levels of the Socioecological Model. This qualitative study is the second part of a larger mixed-method study that converges quantitative and qualitative data. In the quantitative first phase of this study, pre-pregnancy overweight and obesity, diabetes in pregnancy status, and monthly household income were found to be associated with suboptimal GWG, which has been published elsewhere [19]. As the closed-ended nature of the assessment tools used in the quantitative phase of the study was unable to explain these factors, a qualitative, in-depth exploration was conducted based on interpretive philosophy to establish a more comprehensive picture of the phenomenon [20]. Hence, this study aimed to explain the factors associated with suboptimal GWG among pregnant women in Selangor, contributing to the body of knowledge on GWG in general and for developing countries in particular.

## 2. Materials and Methods

### 2.1. Study Design and Setting

This study employed a mixed-methods sequential explanatory design, integrating quantitative and qualitative data to address the complexity of GWG. This approach leveraged the strengths of both methods while compensating for their limitations, enabling a more robust analysis of biological and social factors associated with GWG. The quantitative survey provided a population-level overview, while qualitative inquiries offered context-specific explanations. The study began with quantitative analysis of factors associated with GWG [19], which eventually informed sample selection and guided in-depth interviews in the qualitative phase [21]. The study flowchart is shown in Figure S1.

### 2.2. Quantitative Phase

The aim of the first phase of this study was to determine the proportion and factors associated with suboptimal GWG [19]. A cross-sectional study was conducted between January and March 2020 in Selangor. Selangor is the most developed and populated state (5.46 million) in western Malaysia, with the second greatest population growth rate (2.7%) after Putrajaya (17.8%) and high urbanisation (91.4%) [22]. As such, this state was selected to represent the globalisation of developing countries.

A total of 475 pregnant women from 18 health clinics were selected to participate in this study. A multinomial logistic model showed that, after controlling for confounders, pregnant women with overweight (AdjOR 5.18, 95% CI: 2.52, 10.62, *p* < 0.001) or obese (AdjOR 17.95, 95% CI: 8.13, 36.95, *p* < 0.001) BMI pre-pregnancy were significantly associated with excessive GWG compared to those with normoweight, while women with middle (M40) (AdjOR 2.33, 95% CI: 1.09, 4.96, *p* = 0.029) and low (B40) monthly household income (AdjOR 2.22, 95% CI: 1.07, 4.72, *p* = 0.039), and those with obese BMI pre-pregnancy (AdjOR 2.77, 95% CI: 1.43, 5.35, *p* = 0.002), were significantly associated with inadequate GWG. In Malaysia, the household income was categorised into three groups, namely the top 20 percent (T20), earning ≥MYR 9620; middle 40 percent (M40), earning MYR 4360–9619; and bottom 40 percent (B40), earning less than MYR 4360 per month [23]. Note that 1 USD was equivalent to MYR 4.23 as of 6 May 2025.

### 2.3. Qualitative Phase

Data collection for explanatory qualitative phase was conducted between July 2020 and March 2021. Phenomenology was used as the methodological framework and theoretical approach to describe the essence of a phenomenon by exploring it from the perspective of those who have experienced that particular event [24]. It is both a methodological approach and a theory to view the data. The explanatory aspect of the qualitative phase allowed the exploration of these factors in greater detail to help understand how women experience and navigate the predictive factors. The research question for this phenomenological study was “How do factors associated with suboptimal GWG operationalize among pregnant women in Selangor”?

#### Research Methods

In-depth phone interviews (IDIs) were conducted by the first author, a female public health medical officer with trained interviewing skills and expertise in maternal and child health and nutrition, supervised by NA Hamid and Rohana AJ, experts in the studied field. Interviews were conducted by phone due to the COVID-19 pandemic to protect the vulnerable group from the risk of transmission, compounded by the national Movement Control Order (MCO), which restricted travel between districts. Antenatal visits were limited, and strict physical distancing measures at health clinics prevented in-person interviews, presenting a challenge for face-to-face data collection.

### 2.4. Study Population and Sampling

The participants were selected through purposive sampling among pregnant women from the quantitative phase who had consented to proceed to the qualitative phase if selected. This non-probability sampling method ensured that participants could meaningfully inform the research problem and central phenomenon of the study. Consideration was given to ensure a representative sample across age, gravidity, residence, education level, and employment status, focusing on respondents who were cooperative and willing to share their experiences [21]. Inclusion criteria include those aged 18 and above who were Malaysian citizens who had suboptimal GWG, i.e., either inadequate or excessive GWG. Of the 60 eligible women with suboptimal GWG approached for in-depth interviews, 20 participants (aged 20–37) provided consent. Eight declined due to work or childcare commitments, two were in labor, and thirty were primarily from low-income (B40) households with lower educational backgrounds and were unreachable despite a minimum of three contact attempts. Sampling continued until data saturation, which occurred during the fifteenth interview. Three additional interviews were conducted after saturation was reached to confirm there were no new themes emerged [25]. Thus, a total of 20 respondents were interviewed, including two participants who had incomplete interview sessions due to unforeseen circumstances and could not find the time to be re-interviewed.

### 2.5. Theoretical Framework

The Socioecological Model (SEM) was applied as the theoretical framework for this study to understand the multifaceted and interactive effects of personal and environmental factors on behaviour. The SEM consists of five nested, hierarchical levels: individual (knowledge, attitude), interpersonal (family and friends), community (logistics), organizational, and policy (complex policies). These have been widely used in research on factors influencing gestational weight gain (GWG) in pregnant women [9,10,26]. In this study, SEM guided the research question, data interpretation, and interview guide development.

### 2.6. Data Collection

#### 2.6.1. Recruitment of Participants

Selected participants were contacted via telephone, and those who consented were offered to be interviewed at a time convenient to them [27]. The study’s objectives were explained, and all participants were assured their privacy would be protected by pseudonyms. Informed consent was obtained verbally and audio recorded. The interview sessions varied from 45 to 90 min (average 60 min) in Malay or English. Open-ended, non-sensitive, and broader questions were first asked to establish rapport. Narrow and sensitive questions were asked later, when the participants were more comfortable. All interviewees consented to be audio recorded. Each interview was verbatim transcribed. Field notes were recorded after each interview. Respondents were given honorarium at the end of the session through postage.

#### 2.6.2. Semi-Structured Interview Guide

A semi-structured interview guide was developed based on the first phase’s findings and literature review, with the Socioecological Model (SEM) serving as the framework. The factors related to suboptimal GWG were integrated into the interview guide. It was designed to prompt participants to explain how it affected the weight gain as indicated in the study’s first phase. The content of the finalised interview guide was examined by NA Hamid, an expert in qualitative studies, and refined accordingly. Prior to the actual IDIs, a pilot interview was conducted with two participants (37 weeks pregnant with excessive GWG and 2 months postpartum with inadequate GWG) to assess the feasibility of the study, improve the interview guide, and subsequent sample recruitment [28]. The interview guide was shown in Method S1.

Prior to the actual in-depth interview, a pilot interview was carried out among pregnant women under the supervision of an expert researcher. A pilot interview is a small-scale methodological test used to prepare for a larger study and to ensure that methods work [29]. It was conducted with two participants who were 37 weeks pregnant and 2 months postpartum and had excessive and inadequate GWG to assess the feasibility, improve the interview guide, and subsequent sample recruitment [28]. Based on their feedback, amendments to the questions were made, and future questions were generated.

#### 2.6.3. Ethical Consideration

Ethical approval for this study was obtained from the Medical Research and Ethics Committee (MREC), Ministry of Health Malaysia (NMRR-19-3283-51839), and the Research and Ethics Committee, Universiti Sains Malaysia (JePeM Code: USM/JePeM/19110812). Participation or refusal had no impact on their routine antenatal care.

### 2.7. Data Analysis

Data analysis was performed following the initial interview and continued throughout the data collection phase. The transcribed data were analysed using a six-step thematic analysis process in accordance with Braun and Clarke [30], which involved (1) getting familiar with the data, (2) generating initial codes (descriptive and in vivo coding), (3) searching for themes, (4) reviewing themes (coherence and matched with the predictors from phase 1), (5) defining and naming themes (ensure essence of themes captured), and (6) producing the report. The data were coded and themed before proceeding with subsequent interviews. The coding process followed Saldaña’s five-step method for codifying and categorizing data. It began with line-by-line transcription, followed by labeling meaningful text using in vivo coding. Focused axial coding was then applied to identify the most important codes and classify similar ones into categories. These categories were labeled and grouped to identify higher-order commonalities, known as themes. The data were analysed using Microsoft Excel 2013 to facilitate data management. First author independently coded the transcripts using an inductive approach. This approach was used as it allowed themes to emerge directly from participants’ experiences. The final analysis and results were discussed with the experts. Thematic analysis provides greater flexibility than other methods, which are closely bound to specific theories, allowing for rich, detailed, and complex descriptions of the data. The analysis process was more recursive than linear. To ensure rigor of data, qualitative expert interview supervision, prolonged engagement and regular debriefing with NA Hamid, and methodological triangulation of quantitative survey and IDI findings were used. For evaluation and transferability, samples were recruited with maximum variations in settings, gravidity, education, and employment position. Dependability was ensured by careful transcription in the methodology and analysis of the data. An audit trail comprising raw data, field notes, process notes, and reflective notes helped establish confirmability.

## 3. Results

Table S1 summarised the characteristics of the respondents. Of the 20 participants, most were under 35 years of age (*n* = 16, 80%) and were either multiparous or grand multiparous (*n* = 16, 80%). Almost half had pre-university education (*n* = 8, 40%), followed by secondary (*n* = 7, 35%) and tertiary education (*n* = 5, 25%). More than half were not employed during pregnancy (*n* = 12, 60%). Over half were non-diabetic (*n* = 11, 55%), while the remaining had gestational diabetes (*n* = 9, 45%). Most were in the postpartum period at the time of interview (*n* = 18, 90%), and pre-pregnancy BMI was obese in the majority of participants (*n* = 15, 75%), overweight in 4 (20%), and underweight in 1 (5%). Most had inadequate gestational weight gain (*n* = 15, 75%).

**Figure 1:**
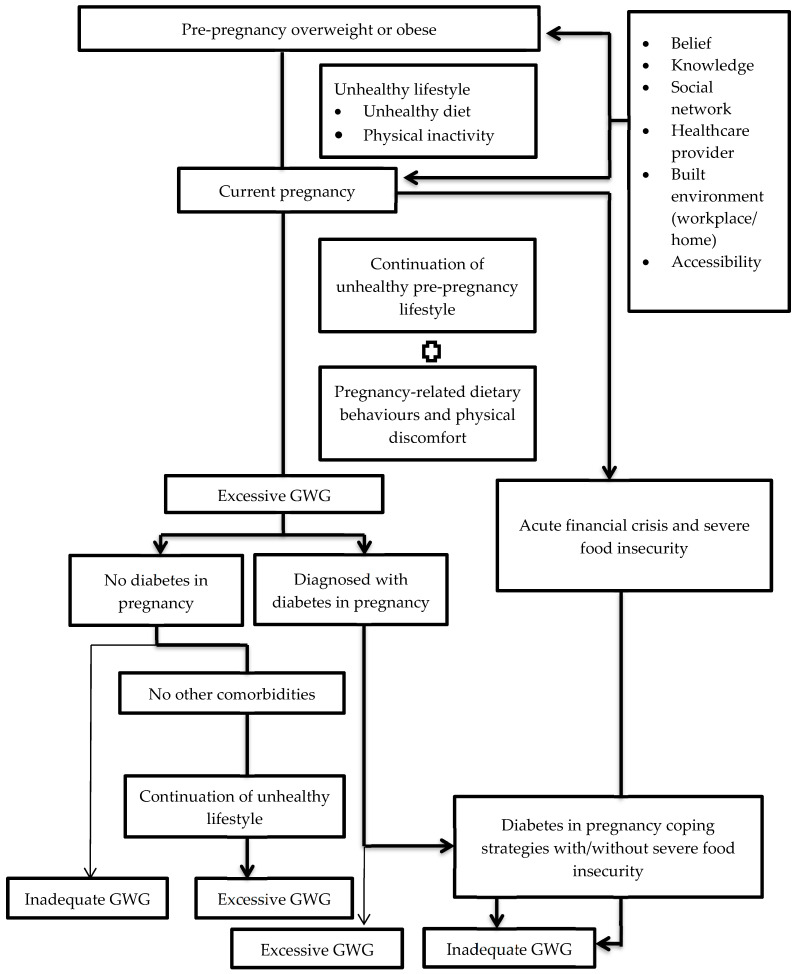
Pathway diagram describing predictors of suboptimal GWG.

The in-depth interviews revealed several categories, which were organized into three key themes: (1) the impact of pre-pregnancy overweight and obesity, (2) managing diabetes during pregnancy, and (3) the influence of middle and low household income. Figure 1 illustrates the interrelation between these themes. Table S2 summarizes the themes and categories.

### 3.1. Excessive GWG

#### Theme 1: The Impact of Pre-Pregnancy Overweight and Obesity

Most participants stated that their unhealthy lifestyles contributed to their weight gain and led to overweight or obese status before pregnancy. This was contributed by (1) a preference for unhealthy diets at pre-pregnancy, (2) a work nature and built environment that promotes unhealthy diets and physical inactivity, and (3) a limited disposable income, which forces participants to purchase cheap, low-quality food.

Most participants favoured diets high in sugar, salt, and saturated fat, often overeating and consuming few fruits and vegetables before pregnancy. They ate high-calorie junk foods like “kerepek” (chips), “cekedis” (snacks), and “asam” (pickled food) alongside three main meals, while others described their pre-pregnancy habits as ’eating a lot’ or ’eating everything’. Some participants, especially multiparous women, gained weight after successive pregnancies, while others blamed contraceptives. R11, para 2, shared her struggle with weight gain before her current pregnancy: “It’s really hard to lose weight (laughs). I didn’t weigh much when I got married—55 kg. After marriage and pregnancy, my weight increased to 90 kg. I started gaining weight after my first baby”. Para refers to the number of times a woman has given birth to a viable infant (≥20 weeks).

Several participants gained weight after starting their work, as disposable income allowed them to buy any food they wished. Stressful or monotonous work environments also led to comfort eating. “When I’m bored, I eat (chips)”, said R3, para 1, who chewed all day while waiting for clients to pay at the cashier. Another participant, R2, para 1, with a busy office job and tight deadlines, said, “When I’m stressed out, I eat (chips)”. Some participants perceived their attempts to lose weight as failures, leading to frustration and continued overeating.

Long work hours, weekend shifts, and tight deadlines influence participants’ eating habits, leading to late dinners, skipped meals, unbalanced diets, and unhealthy office snacking. Many also consumed fast or junk food late at night while working overtime. Jobs in clerical, supervisory, managerial, and benchwork roles encourage sedentary behaviour. Hectic schedules, fatigue, and reliance on physical activity at work were common reasons for inactivity before pregnancy. Participants viewed workplace environments, particularly in urban areas, as obesogenic due to the abundance of high-calorie, salty, sugary, and fatty foods from nearby fast food outlets and food stalls, which they frequently purchased during breaks or on their way home. Also, some participants’ neighborhoods were surrounded by an abundance of eateries, enticing them to purchase food.

Participants described their eating habits during pregnancy in various ways. Most overweight or obese participants continued their unhealthy pre-pregnancy eating habits. As R15, para 4 stated, “I eat exactly like when I wasn’t pregnant. I’ll eat whatever I want”. Several participants reported that their overeating habits worsened during pregnancy. R5, para 1 mentioned, “I was a big eater before (pregnancy), but I believe it doubled during pregnancy”. Changes in food preferences were also linked to cravings, with most participants who gained excessive weight reporting cravings for sweet items. These participants attributed cravings to a phenomenon that is known as “pembawakan bayi” or “fetal influence”. R5, para 1, explained, “Whatever we feel like eating, it’s not from us (the mother), it’s the baby. The baby wants to eat it”, and that the desire to consume specific foods only exists during pregnancy. If these cravings are not fulfilled, something unpleasant will occur (e.g., the baby will drool), and this belief has been passed down through generations. Some participants believed the type of food that leads to women’s cravings was attributed to the baby’s gender during respective pregnancy, craving sweet food if the baby was a girl and meat if the baby was a boy. Others overindulged in sweets to alleviate nausea and vomiting in pregnancy. Many participants indicated that they could only tolerate fast food, outside meals, and sweet desserts (e.g., chocolate cakes, chocolate ice cream, and strawberry-flavoured milk) to satisfy their hunger during this period, resulting in rapid weight gain.

As previously stated, most overweight and obese women led sedentary lifestyles before and during pregnancy, contributing to excessive GWG. Pregnancy-related discomforts, such as breathing difficulties, swollen feet, soreness, and morning sickness, further aggravated this lack of physical activity. Some participants admitted to being too lazy to exercise, while primigravida women, still adapting to pregnancy, reported feeling “not used to it yet” and becoming easily fatigued. Despite understanding the benefits of physical activity for easing labor and maintaining fitness, most participants felt too fatigued to engage in exercise. Pregnancy fatigue and the “heaviness” of their body weight caused several women to abandon exercise after a few attempts. This was worsened by time constraints due to work and personal obligations (i.e., childcare), particularly among multipara women.

Some participants attributed excessive eating and GWG to specific phases or events during pregnancy. Several women reported overeating and excessive weight gain in the third trimester, feeling obligated to overeat before delivery, knowing they would need to restrict their diet during confinement. The impact of the phase 1 Movement Control Order (MCO) aimed at containing the COVID-19 pandemic was discussed. The MCO restricted travel, social gatherings, and the operation of non-essential industries, businesses, educational institutions, and government offices. Most overweight or obese participants reported overeating and excessive snacking during this period, with some resorting to junk food. Emotional eating, driven by boredom from staying home, led many to spend more time cooking and consuming frozen, convenient foods. As a result, most participants experienced significant weight gain due to a sedentary lifestyle during the MCO.

“Yes, I ate a lot during the MCO. Everyone did. I ate constantly and quickly ran out of money without realizing it. I cooked all the time—cooking and eating. I bought frozen food and ate frozen curry puffs and spring rolls. Almost every evening, I ate until night time. I weighed 97 kg, almost 100 kg at that time”. (R8, para 4)

Participants’ diet and physical activity habits were influenced by their individual characteristics and surroundings, including pregnancy-related beliefs, social networks, work environments, healthcare providers (HCPs), and financial circumstances. Overweight or obese participants who gained excessive weight often believed overeating and weight gain were the norm and accepted weight gain as an unavoidable part of pregnancy. While many participants reported uncontrollable appetites, some attributed this to hormonal changes. Most believed cravings, aversions, hunger, uncontrollable appetite, and fatigue were the baby’s way of communicating. When asked why they over-ate and consumed high-calorie, sugary, and fatty foods, several stated they did so to ensure the baby received sufficient nutrients.

Family members’ overprotective behaviour, driven by concerns for the baby’s health and safety, was discussed. Most participants reported that their husbands and family members frequently pampered them with food to ensure the baby received adequate nutrition. Family members often provided participants’ favourite foods, including unhealthy, high-calorie processed items, which contributed to excessive weight gain during pregnancy. Although some participants were unable to cook due to nausea and vomiting during pregnancy, their spouses ensured food was always available. The majority felt pressured by their close social network, especially spouses, in-laws, siblings, and friends, to “eat for two” to feed the baby. As a result, they struggled to maintain a healthy GWG. Despite knowing they did not need to overeat, some participants found it difficult to resist family pressure to eat more. R5, para 1, described being pressured to eat more and later at night for the baby, despite her desire not to, quoting her father-in-law: “You can eat at 8 p.m., but you must eat again after Isha prayers (8.30 p.m.). If not, the baby will be hungry. Don’t restrict eating, it’s not good”.

Moreover, family members, especially spouses, often prevented physical activity (PA) by removing active tasks, citing concerns for the baby’s safety or to relieve participants of household chores. The most common reason for limiting activity was fear of miscarriage, especially for a “precious baby” or due to previous miscarriages in the family after PA during pregnancy. Several participants stated that, despite being active before pregnancy, they stopped exercising at their spouse’s or mother’s advice, fearing it could harm the baby—such as causing the umbilical cord to wrap around the baby’s neck. Overprotective behaviours were more prevalent among primigravidas, those expecting the first grandchild, or those carrying “precious babies”. R5, para 1 described how her older husband, who married late and was expecting their first child, became overprotective, quoting him, “He married late. So, when he found out I was pregnant, he told me to take good care of the pregnancy”.

Several participants highlighted the importance of social support, particularly from spouses and family, in influencing GWG. Spouses were often cited as dining companions, especially during late-night meals. Many overweight or obese participants who were employed reported that co-workers with similar eating habits and preferences often reminded and encouraged each other to eat, leading to excessive calorie intake and weight gain. Shared meals were described as enjoyable, and this continued into pregnancy. Some participants attributed inactivity during pregnancy to a lack of companionship from their spouses, which they believed contributed to difficult deliveries. R14 (para 1) said, “(I know) exercising is important, but I was bored, so I didn’t [laughs]. I was alone and bored because I didn’t have anyone to accompany me… my husband works the morning shift from Monday to Sunday with no days off”. This participant also suggested that health clinics provide affordable gyms for pregnant women, offering opportunities for social interaction and discussion.

Participants who attempted to lose weight before pregnancy was motivated by the desire to “lose weight before marriage or pregnancy”, “partner pressure”, and “body image”. However, all of them stopped their weight loss efforts upon becoming pregnant, viewing pregnancy as a time for more positive body image and a temporary reprieve from negative comments.

All pregnant women reported receiving antenatal care and weight monitoring at government or private clinics. Overweight and obese participants reported that their HCPs addressed GWG in varying ways. Some were reassured their weight gain was “fine” and not to worry despite gaining excessively. R2, para 1 shared, “She (the nurse) said, ‘You gained a lot of weight, but it’s okay, it’s not that bad. It’s normal. You will lose the weight after childbirth’. But for me, I had gained 20 kg. It was terrible”. Others received little to no conversation on GWG, assuming all was well if their HCPs did not address it. R5, para 1 stated, “Since I wasn’t referred to a doctor, I believed I was still well. If it was urgent and dangerous, I would have been referred to a doctor, right”?

Many participants felt HCPs prioritised other medical issues over GWG discussions. Most also highlighted minimal advice on exercise during pregnancy, often limited to vague suggestions like “do the exercise” without explaining its benefits or relevance or how to perform it specifically, which led many to view exercise as optional and unimportant.

Participants stated that busy clinics with high patient loads affected the quality of information provided. As R5, para 1 explained, “I couldn’t ask because the doctor was busy. I understand; there are so many patients”. Overweight and obese participants often felt judged during weight discussions. Some reported being admonished for “not controlling their diet” when gaining excessively and judged for “not eating” when losing weight. They described such interactions as traumatic and guilt-inducing, particularly during the emotional sensitivity of pregnancy. Past insensitive remarks and inadequate advice prompted several participants to seek information independently, often through online searches. Others ignored their weight gain entirely, as R20, para 2 stated, “I assumed no matter how much weight I gained or lost, I would be admonished. So, I just didn’t bother [laughs]”. Some controlled their diet before appointments to avoid reprimands, while most viewed such feedback as part of HCPs’ duties.

Several participants described access to healthy diets and physical activity as influencing GWG. While the majority of respondents felt that fruits and vegetables were inexpensive, others felt that they were perishable and costly. R6, para 1 noted, “Now that I’m married, I don’t accompany my spouse to the market if only RM10 is available because it’s not enough. If I go to the market, I spend RM50 on fruits alone”. Certain foods, including seafood, whole bread, maternity milk, and some fruits, were considered costly, limiting purchases during pregnancy. Although aware of the nutritional value of healthy food, many women were forced to split purchases with family members due to high costs. Some participants also highlighted concerns about their family’s acceptance of healthier meals and the added expense of preparing two separate meals.

Several overweight and obese participants stated that fluctuating household food supplies led to unhealthy eating patterns before and during pregnancy. Limited finances often forced them to compromise on meal quality, opting for the cheapest foods, which were typically high in calories and fat. R2, para 1 shared, “I asked my husband how much money he had today, and he said RM10. When I asked him what we were going to eat, he said, ‘We just eat burgers’”. These participants faced increased risks of severe food insecurity and inadequate GWG during financial crises, as discussed in the next section.

As illustrated in Figure 1, overweight and obese participants without comorbidities that caused food aversion, dietary restrictions, or lifestyle changes often maintained unhealthy eating habits and physical inactivity during pregnancy, resulting in excessive GWG, as they viewed themselves as ‘healthy’. However, those with comorbidities, such as severe hyperemesis gravidarum (a severe form of nausea and vomiting leading to dehydration and weight loss), diabetes, food allergies, or fibroids (non-cancerous uterine growths), were more likely to report inadequate GWG due to dietary restrictions. Participants with fibroids avoided foods like chicken, fish, and vegetables due to taboos, relying instead on eggs and potatoes. Severe HG left some consuming only porridge and anchovies, leading to anaemia and low-birth-weight (LBW) babies. Others resumed pre-pregnancy diets due to misunderstandings about nutritional needs or workplace challenges, such as being too busy or unable to find nearby eateries, which led to skipped meals. Some limited their food intake to avoid repeated glucose tests (MOGTTs) after excessive GWG.

### 3.2. Inadequate GWG

#### 3.2.1. Theme 2: Managing Diabetes During Pregnancy

Most participants reported that having diabetes during pregnancy affected their gestational weight gain (GWG) in several ways. First, the diagnosis served as a wake-up call. Second, it had an emotional impact due to its effects on maternal and feotal health. Third, the diagnosis led them to adopt extreme dietary coping strategies to prevent weight gain. Fourth, it caused pregnant women to prioritize the health of their babies over their own. Fifth, healthcare providers (HCPs) advised focusing on diabetes control rather than maternal nutritional status. Lastly, there was a long waiting period for a dietitian’s appointment.

Nearly all participants viewed their diabetes diagnosis during pregnancy as a wake-up call. Most reported having a pre-existing unhealthy diet high in calories, sugar, and fat, along with a sedentary lifestyle. These behaviours were exacerbated during pregnancy due to hyperemesis gravidarum (HG), cravings, and physical discomfort, contributing to significant weight gain in the first and second trimesters prior to diagnosis.

“[…] because I enjoyed cake before I had diabetes, that’s the reason (I got diabetes) [laughs]. Cake and Dutch Lady strawberry milk. I can go a day without rice. All I’ll have is cake and milk because of the morning sickness. Then, five months into my pregnancy, I was diagnosed with diabetes. Since then, I’ve started to control (my diet)”. (R17, para 2)

Several participants attributed their diagnosis to unhealthy dietary practices within their families, pre-pregnancy obesity, and a sedentary lifestyle during the Movement Control Order (MCO). R1, para 5, a housewife, explained that her previous role as a canteen assistant helped her stay active despite being obese, but the MCO led to a more sedentary lifestyle: “The MCO prevented me from going anywhere. I’m only eating, sleeping, and resting at home. Not much housework, no sweating, and I felt less active. Then I’m always eating. That’s one of the causes (of having diabetes)”.

Upon being diagnosed with diabetes, most participants reported feeling ‘fear’, ‘anxiety’, ‘confusion’, ‘uncertainty’, ‘frustration’, ‘self-blame’, ‘stress’, ‘feeling down’, and ‘depressed’. This response was most common among women experiencing their first pregnancy with diabetes. Many participants wanted a ‘normal’ pregnancy like other women and voiced concerns about pregnancy complications from uncontrolled diabetes, such as miscarriage, large or malformed babies, delivery difficulties, and intrauterine death. Some worried about persistent diabetes due to family history. Overthinking the effects of gestational diabetes mellitus (GDM) negatively impacted participants’ moods, and some resulted in significant appetite loss. Several also struggled with severe hyperemesis gravidarum (HG) and food allergies, while others blamed hematinic medications and metformin for reducing their appetites and limiting food intake.

The worry, fear, and stress accompanying the diagnosis strongly motivated most participants to adopt healthier lifestyles, including switching to foods with low glycaemic indices (e.g., brown rice and oats), reducing meal portions and frequency, restricting carbohydrate, sugar, and fat intake, and synchronizing the timing of medicine and food intake. They used a ‘trial and error’ approach to find a diet that suited them.

Upon further probing, some participants reported using extreme coping mechanisms to manage their blood glucose levels (BGLs). R20, para 2, described her excessive dietary restrictions: “Sometimes, I’d just eat rice with vegetables. At other times, I’d only eat fish” Others fasted to the point of losing weight during pregnancy. One participant said, “I fasted a lot while pregnant. I didn’t eat much during my fasts, just Jacob’s milk crackers the whole day. I lost weight” (R9, para 2). Some skipped main meals, eating only once a day and often feeling hungry. One participant mentioned, “I eat once a day. That’s enough. I skip dinner. If I’m hungry or tired at night, I eat biscuits” (R11, para 2). Others avoided snacking and reduced carbohydrates by abstaining from rice entirely. R1, G5P4, stated, “I didn’t eat rice at all! [excitedly] I only ate wholemeal bread and vegetables. That’s why I lost weight”.

Despite contradicting healthcare providers’ (HCPs’) advice, many participants adhered to strict dietary regimes that they felt better regulated their blood glucose levels (BGLs). R17, para 2, explained, “The doctor told me to eat rice twice a day, but I only ate it once because I was afraid my blood sugar profile (BSP) would rise”. R20, para 2, shared similar concerns. BGLs, therefore, became the cornerstone of their food choices rather than the calorie recommendations from dieticians. As a result, weight loss continued despite good glycaemic control. R4, G1P0, recounted, “My sugar was under control, but I continued losing weight and never regained it. I went from 97 kg to 89 kg”.

Furthermore, some participants feared the initiation of medications, particularly insulin, if they were not able to maintain normal blood glucose; others were reluctant to commit to more frequent clinic visits and perceived medical treatment as a punishment for not controlling their diet. As a result, they made every effort to keep their BGLs under control, indicated as ‘non-red’ on their BSP monitors, to avoid being prescribed insulin. One participant noted, “I was worried I’d need medicines or insulin. […] Sure, I was hungry [laughs], but I was terrified, so I dieted until I went from 90 kg to 70 kg” (R4, primigravida).

Those who controlled their diet reported higher stress levels and became more obsessed with dietary restrictions compared to participants on medications. The latter group, who accepted their diagnosis, were less likely to restrict their diets. Some participants, however, reduced their food intake only on the days of their BGL monitoring, finding it difficult to control their food consistently. These participants were more likely to experience excessive weight gain. As R20, para 2, explained, ”I kept eating sugary and sweet things I shouldn’t have. Even though I ate them, my blood sugar was okay, probably because I was on medication. It’s tough because I always want to eat!”

Most participants with diabetes expressed greater concern for the health of their baby over their own health or nutritional status. This was seen in both pre-existing diabetes and newly diagnosed GDM. The extreme coping strategies that several participants adopted were spurred by a false sense of security that foetal health was not compromised as it continued to gain weight normally, no observable abnormalities were detected during ultrasound examinations, and their BGLs were well controlled despite their weight loss. Some participants indicated that gaining weight during pregnancy was unnecessary and that maintaining optimal glycaemic control was more important than losing weight. They were unconcerned with their weight loss as long as their baby was growing well. As R4, para 1 put it, “*I didn’t mind if I lost weight. I’d be more worried if the baby lost weight. But when I was scanned at the hospital, the doctor said that the baby’s weight was fine and that there were no issues. So I was OK*”. These participants viewed gaining less weight or losing weight during pregnancy as an indirect benefit of having diabetes. They were delighted to reach a normal BMI during pregnancy as they had struggled to lose weight pre-pregnancy. This further reinforces their dietary restrictions. R8, para 3, said, *“It’s okay (the weight loss) because I intended to lose weight anyway”.* Furthermore, although they were concerned that the baby did not have adequate nutrition, several overweight and obese participants voiced skepticism about gaining more weight when counselled by the HCPs. R16, para 1, said, “*The nurse told me that I need to gain more weight. Then I thought, ‘Do I need to put on more weight? I’m already heavy. Don’t tell me I need to reach 100 kgs?’ I did worry if my baby wasn’t gaining enough weight*”.

Participants highlighted that healthcare providers (HCPs) prioritized diabetes control over maternal nutrition. Some recounted inconsistent advice on inadequate weight gain among pregnant women with diabetes. Several mentioned that some HCPs considered weight loss normal with healthier dietary changes, as long as it was not drastic, while others raised concerns. Some participants felt that GWG was not a priority, with HCPs focusing instead on critical health issues like diabetes and anemia. As R7, para 4, said, “My weight? Not really. They mainly focused on my diabetes. They didn’t review my weight. What’s for sure is it’s all about my BGL. They only wanted to know about my BGLs”. Since HCPs did not address poor weight gain, participants assumed it was not a concern, reinforcing their excessive dietary restrictions and continued weight loss.

Busy clinics and prolonged waiting periods for appointments contributed to inadequate gestational weight gain (GWG) among participants with diabetes. Several participants reported that initial feelings of fear, low mood, and uncertainty led them to over-restrict their food intake in an attempt to quickly control their blood glucose levels (BGLs) while waiting for a dietitian appointment. This affected their weight gain. R4, a primigravida, stated, “I was devastated when I found out I had diabetes. It took a long time to get an appointment with the dietitian. I waited almost a month to see one”. She added, “At that time, my BGLs were high and uncontrolled. This made me more cautious with my diet, so I ate less and started losing weight”. Several participants delivered before meeting with a dietitian, either due to pregnancy complications or rescheduled appointments. Others noted that dietitian services were temporarily suspended during Phase 1 of the Movement Control Order (MCO). Participants emphasized the importance of meeting with a dietitian, as it allowed for two-way communication, unlike online resources. Additionally, due to time constraints, other healthcare providers (HCPs) were unable to offer the same level of comprehensive dietary guidance as dietitians.

#### 3.2.2. Theme 3: The Influence of Middle and Low Household Income Status

Financial hardships due to multiple commitments, high living costs, and the additional expenses of preparing for the arrival of a new baby left participants with limited disposable income for food. Participants highlighted various financial obligations, including childcare (e.g., nursery and school fees), housing or vehicle loan repayments, education loan payments, summons, rent, utilities, groceries, and transportation. Many working participants also spent more on dining out due to a lack of time to cook.

Some participants faced additional work-related expenses, such as high commuting costs, which exceeded their earnings, causing some to resign. They struggled to balance limited budgets with baby-related expenses. As R2, M40, said, “My income is fully used for commitments. At the end of the day, I have only RM200 or RM300 left to save for the baby”.

Participants with comorbidities, such as diabetes, incurred extra costs for regular BGL monitoring and low-GI foods, which are more expensive. Some could afford glucometers, while others had to pay for night glucose monitoring at pharmacies. Maintaining stable BGLs became a constant challenge that impacted their time, work, and income. One participant on intensive insulin treatment explained, “My salary was cut because I had to take unpaid leave every week for BGL monitoring”, further straining the household finances.

Several participants reported working multiple part-time jobs and sharing household responsibilities with spouses or family members, such as parents or siblings, to alleviate financial stress and save extra income to prepare for a newborn. By working around the clock, these responsibilities included dividing the responsibility to pay off utilities, rent, food, and childcare. One participant, R4, an M40, who shares a rented house with her brother, explained, “When I don’t have money for food, my brother buys it for me. When I get paid, I buy food for him”. Another participant briefly operated a side business to generate additional income but discontinued it due to fatigue during pregnancy and time constraints.

When it comes to grocery shopping, the majority of participants compared prices across multiple markets avoided premium stores and purchased smaller, more affordable items. Due to budget constraints, some prioritized essential baby products. R14, an M40, stated, “When I was seven months pregnant, I panicked and bought essential items like baby clothes, baby binders, and only a mattress for the bed”. Many participants noted that they were taught to purchase inexpensive items by family members, particularly those from lower-income households, and, in turn, taught their children to consume whatever was available and choose the least expensive, often nutritionally inadequate, meals to save money.

Pregnancy increases vulnerability to job instability, as pregnant women are more likely to leave their jobs. Participants reported difficulties maintaining employment due to pregnancy-related discomfort, comorbidities requiring regular check-ups, and the nature of their jobs, such as heavy workloads, stress, long commutes, and childcare responsibilities. In addition to hyperemesis gravidarum (HG), concerns about the unborn baby’s safety due to the nature of their work were frequently cited as reasons for quitting. Most participants stopped working during the first trimester.

R15, a B40 who resigned due to severe HG and diabetes requiring insulin, explained, “I finally gave up and resigned from my job… My morning sickness made it difficult to work”. Another participant, R4, an M40, attributed her decision to quit to the long 3 h commute, which caused fatigue and drowsiness while driving, as well as work-related stress that impacted her pregnancy. She stated, “My workload increased, and my blood pressure started to rise during check-ups, probably due to stress at work. After I quit, my blood pressure normalised”. Several multiparous participants mentioned resigning after the birth of previous children, with the most common reason being the lack of childcare, particularly for sick children. This situation placed a financial burden on the household, as the responsibility for providing basic needs shifted entirely to the husband.

Additionally, pregnancy-related discomfort and comorbidities limited participants’ ability to cook, which was further exacerbated by insufficient funds for meals. R8, a para 2, M40, who has diabetes, shared, “I’d want to buy food, but I’d find that I was short on money. I’d want to cook, but I’d be unwell. I’m just exhausted. I’d want to feed my children, but I’m too tired to cook”.

However, some participants indicated that resigning while pregnant did not significantly affect them due to strong familial support. R13, from a top-income (T20) household, living with her parents, who provided for basic household needs, stated, “It’s usually my parents who buy the groceries. My mother works, and my father is retired. Since I live with my parents, I’m not really affected (by quitting my job)”.

Job insecurity and employment loss heightened vulnerability to severe food insecurity, particularly when the primary breadwinner, typically the husband, lost his job during the COVID-19 pandemic. Several participants shared their own and their spouses’ experiences of salary cuts or no salary due to temporary business closures during Phase 1 of the Movement Control Order (MCO). Others quit working due to concerns over the impact of a potential COVID-19 infection on their partner’s pregnancy. Lorry drivers and factory workers were frequently cited as particularly affected, as they did not receive pay during the MCO. R17, a B40 participant, explained, “My husband usually gets paid monthly, but since the MCO began, he gets paid daily. He’s only called if there’s work. He hasn’t been hired in a month, so it’s affecting our income. I had quit working because of pregnancy”.

Another participant was asked to stop working temporarily due to her pregnancy, and her salary was halved before being fully suspended during the MCO. As a result of job loss or reduced wages, some participants reported prioritizing their limited finances to meet household obligations. One participant, who already had a restricted food budget before the pandemic, found it even more constrained during the MCO. The majority of participants reported eating whatever was available due to limited finances, with eggs, flour-based dishes, and canned foods being common staples. Despite these hardships, all participants insisted that they would prioritize feeding their children over themselves or their partners. To ensure their children had enough to eat, several participants ate less or skipped meals, enduring hunger to save food for the next meal.

To cope with food scarcity, some participants borrowed money or sought food from family members, while others were too embarrassed to ask for help. Several expressed concern about not having enough food to sustain themselves, their unborn baby, and their children during pregnancy. Short-term, irregular aid and long wait times further disrupted food security. However, most participants highlighted that assistance from the government’s COVID-19 relief programs (e.g., funding, loan moratoriums, and food support), non-governmental organizations (NGOs), and individuals alleviated food scarcity and “eased the burden”. This aid benefited both the B40 and M40 groups, many of whom faced sudden job losses or salary reductions.

## 4. Discussion

The quantitative phase of this study identified factors associated with suboptimal gestational weight gain (GWG) [19]. Subsequently, follow-up in-depth interviews with a diverse group of participants who experienced suboptimal GWG provided further explained the context for the quantitative findings. Socioecological Model (SEM) was used to examine the factors at multiple levels, highlighting the dynamic interplay between individuals and their environment. Three key themes emerged from the qualitative phase that aligned with the quantitative results: (1) the impact of pre-pregnancy overweight and obesity, (2) managing diabetes during pregnancy, and (3) the influence of middle and low household income. A pathway was developed to illustrate the explanatory pathway of suboptimal GWG.

Being both overweight and obese pre-pregnancy was a significant factor contributing to suboptimal gestational weight gain (GWG). The subsequent qualitative exploration revealed that participants who were overweight or obese before pregnancy tended to have unhealthy lifestyles, including excessive consumption of calories, salt, and sugar, along with physical inactivity. The quantitative phase found that most respondents (59.8%) were employed during pregnancy, while the qualitative phase highlighted that the majority of these participants attributed the obesogenic workplace as the primary setting for weight gain before pregnancy, which was further exacerbated during pregnancy. The availability and accessibility of the urban food environment, which promotes overweight and obesity, aligns with findings from a qualitative study in Korea [31]. Long working hours, job-related stress, and sedentary jobs influenced unhealthy eating behaviours, such as meal skipping followed by overeating, the consumption of fast food, and the emotional eating of junk food [32,33]. Studies have shown that emotional eating increases cravings for snacks and hyperpalatable foods, often at the expense of nutritious options, and is strongly associated with a higher intake of calories, fat, carbohydrates, and protein [34,35]. Furthermore, overindulgence in food, especially in the third trimester, was driven by anticipation of confinement restrictions [36]. Factors such as boredom, excess discretionary cash from the moratorium, and the availability of food delivery services, along with appealing sales promotions and marketing strategies, encouraged bulk purchasing, constant eating, and inactivity during the MCO.

Rahman [37] observed that, during the MCO, Malaysians prioritized long-shelf-life and frozen foods so-called ultra-processed food, often at the expense of perishable items. Given the restricted GWG range for overweight and obese pregnant women, excessive calorie consumption led to excessive GWG, with some participants developing gestational diabetes mellitus (GDM). This issue was particularly prevalent among participants whose employment status and financial situation were not significantly impacted by Phase 1 of the MCO.

Further exploration revealed that these unhealthy pre-pregnancy lifestyles were aggravated by pregnancy-related dietary behaviour changes and physical discomfort and were compounded by pregnancy-specific beliefs, social network influences and pressure, healthcare providers’ lack of time and inconsistent GWG advice, higher prices for nutritious food and antenatal courses, and fluctuating household food supplies during pregnancy. All of these promoted excess calorie consumption, physical inactivity throughout pregnancy, and excessive GWG. Pregnancy-related dietary behaviour changes included an exacerbation of pre-pregnancy excessive calorie intake, sweet cravings, or unhealthy food consumption due to hyperemesis gravidarum (HG), as these were the only foods that could be tolerated by the participants. Previous studies have reported similar findings, noting that women with HG crave high-calorie, carbohydrate, fat, and sugar-rich foods to alleviate symptoms [38,39]. According to Blau and Orloff [40], cravings for fatty and fast foods account for nearly 11% of the variance in excessive GWG. Furthermore, as indicated by several of the study’s overweight or obese participants, excessive GWG early in pregnancy increases the risk of gestational diabetes mellitus (GDM) and feotal macrosomia [41].

Despite being aware of the importance of physical activity (PA), pre-pregnancy inactivity due to time constraints related to childcare or work, along with pregnancy-related physical discomfort (i.e., swollen legs, pain, discomfort, tiredness), exacerbated inactivity, leading to excessive GWG. Most participants reported that household chores constituted their PA during pregnancy. This finding corroborated the study’s quantitative results, which indicated that the most prevalent forms of PA were household chores and caregiving, followed by inactivity. Nevertheless, as dieting is not recommended during pregnancy due to concerns that it may affect the unborn baby’s health, the National Institute for Health and Care Excellence (NICE) recommends that overweight or obese pregnant women engage in at least 150 min of moderate-intensity aerobic PA per week to maintain optimal GWG [42].

Moreover, based on the Socioecological Model (SEM), at the intrapersonal and interpersonal levels, pregnancy-specific beliefs and pressure from close social networks were largely driven by the desire to protect and provide the best for the unborn baby [43]. Overprotective behaviours were more pronounced among primigravida, as their families often overindulged them with food, alleviated household chores, or prevented strenuous activities [44]. Consequently, social acceptance indirectly alleviates women’s pressure to gain excessive weight [10]. The need to conform to norms of good mothering, a powerful social construct, also drove pregnant women to adhere to culturally desirable and socially acceptable norms and ideals [45,46]. Most multipara maintained their pre-pregnancy dietary habits to some extent. At the organizational level, healthcare providers (HCPs) were the most trusted source of information. However, the clinic’s busy schedule and time restrictions affected the quality of health information provided, particularly on the sensitive and highly stigmatized topic of weight. Insensitive weight-related remarks, especially toward overweight or obese women, often led to non-compliance with GWG advice [47]. Phase one findings indicated that not receiving nutritional counseling was significantly associated with excessive GWG in univariate analysis. Thus, qualitative findings demonstrated that overweight and obese women relied on their HCPs to address weight gain issues. A lack of knowledge, combined with multiple informal sources of GWG advice, led to confusion and misconceptions, especially among primigravida due to inexperience. When advice was inconsistent or too general, lacking specific strategies for nutrition and exercise, GWG was often perceived as unimportant, resulting in the continuation of unhealthy pre-pregnancy dietary habits, inactivity, and excessive weight gain [43]. Haruna and Yeo [48] reported that HCPs often raised the issue only after excessive GWG had occurred or when prompted by patients. This represented a missed opportunity to prevent excessive GWG, especially among women at risk (overweight or obese pre-pregnancy), as knowledge is a modifiable factor in preventing excessive GWG [49]. Therefore, discussing weight gain during the first trimester is crucial for helping pregnant women manage pregnancy-specific dietary changes (cravings, HG) and physical discomfort [50]. At the policy level, food prices influence food choices. Overweight and obese participants considered healthy diets (e.g., seafood, poultry, fruits, brown rice, and maternity milk) to be expensive, limiting their purchasing power during pregnancy and leading them to rely on less nutritious food. The impact of fluctuating food supplies on binge eating disorders and subsequent excessive weight gain has been reported in previous studies [51,52]. Unlike in Western countries, where cheap fast food drives over-consumption among low-income groups, in which portraying participants from M40 and B40 households in Malaysia tended to favour cheaper street foods with a low diet quality derived from ultra-processed food such as fast food, which contributed to higher consumption of fats, trans fats, salt, and sugar, leading to weight gain [53,54]. This trend is compounded by the higher cost of fast food in Malaysia [55]. The inverse relationship between energy density (MJ/kg) and energy cost results in higher caloric intake per dollar, which contributes to excessive energy intake and, subsequently, weight gain [56], particularly among low-income and less-educated populations, thus exacerbating health inequities [57]. Additionally, the inability to participate in prenatal exercise classes due to high expenses also contributes to excessive GWG.

Unhealthy pre-pregnancy diets and inactivity were maintained by pre-pregnancy overweight or obese women who did not develop comorbidities that would cause food aversion or require dietary adjustment or restriction, resulting in excessive GWG. In the quantitative phase, pre-pregnancy obesity and diabetes were significantly associated with inadequate GWG. Subsequent qualitative exploration revealed that the diagnosis of diabetes served as a wake-up call, highlighting the unhealthy lifestyle habits before and during pregnancy. Pregnant women with GDM had a considerably higher sugar intake ratio in early pregnancy [58] and a lower healthy diet score compared to non-GDM obese pregnant women [59]. These women were most psychologically vulnerable at the time of diagnosis [60,61], which led them to adopt extreme coping strategies, including severe food restriction, fasting, and imbalanced meals that resulted in poor weight gain. Fear of feotal risks due to uncontrolled diabetes, avoidance of medical therapy (particularly insulin), which some regarded as punishment for failing to control their dietary intake, and a desire to avoid additional clinic visits after starting insulin therapy took precedence over concerns about adequate nutrition [62].

Often, rather than focusing on nutritional needs, dietary management was based on achieving a “non-red” (normal) blood glucose level (BGL) record. Since foetal health was seen as the benchmark of overall pregnancy health, women believed their weight loss did not compromise the baby, as antenatal scans showed a normal and growing foetus. This demonstrated a profound lack of awareness regarding the potential risks of inadequate GWG, even though most participants had good knowledge of diabetes-related complications during pregnancy. While most diabetic women gained inadequate weight [63], HCPs placed greater emphasis on glycemic control, which undermined GWG. This gap in GWG counselling led participants to prioritize diabetes control over appropriate nutrition. The extended waiting time for dietitian consultations further perpetuated extreme dietary restrictions. This condition could lead to permanent, deleterious effects on feotal metabolism, altering feotal physiology and function due to insufficient or inappropriate dietary intake in utero (feotal development adaptation), which predisposes the foetus to multiple metabolic, endocrine, cognitive, and cardiovascular disorders later in life [64]. Additionally, pregnancy increases the risk of starved ketosis due to physiological changes in glucose metabolism essential for feotal growth and development [65]. Since pregnant women require 33 g more carbohydrates than non-pregnant women for foetal brain development and function, excessive calorie restriction of fewer than 1500 calories per day (a 50% restriction) induces ketonuria and ketonemia. The risk is amplified by GDM [66] and HG-induced insufficient caloric intake during pregnancy [67]. It is reported that high levels of β-hydroxybutyrate resulted in lower mental developmental index scores and average Stanford–Binet scores in offspring aged two to five years [68]. Given that GWG for diabetic pregnant women aligns with the IOM 2009 recommendations (based on pre-pregnancy BMI), early intervention is critical to avoid ketosis caused by extreme calorie restriction [69].

In terms of income, there was a significant association between low (B40) and middle monthly household income (M40) with inadequate GWG. Subsequent qualitative analysis showed that household income influenced inadequate GWG through, first, multiple commitments and high living costs, which resulted in limited disposable income for food. Although women in the M40 income group earn more than those in the B40 income group, high living costs and multiple commitments—such as mortgage, rent, childcare expenses, utilities, and transportation costs—exacerbated by expenses for newborn preparation, limited both groups’ discretionary cash for food and other necessities [70]. Despite being one of Malaysia’s wealthiest and most populous states, Selangor, as our study locality, comprised one of the highest monthly living expenditures at MYR 3589 for a single household [22]. Additionally, Selangor raised its poverty income threshold by MYR 700 above the national standard to more accurately reflect its high cost of living. Fluctuate income growth, overpriced housing, and rising household debt all contribute to high living costs [71]. Although the national median monthly household income increased by 3.9% and the national median monthly disposable income rose by 4.2% in 2019, household debt continues to rise and has reached high levels [72]. Consistent with the study findings, the World Bank [73] reported that most B40 debt was used to support life commitments (vehicles or personal loans) rather than asset creation. Furthermore, food prices in Malaysia rose compared to other Consumer Price Index categories, which led to household food insecurity. Given that B40 (18.6%) and M40 (14.2%) households spend more on food than T20 households (11.3%) [22], these households are disproportionately affected by the increased inflation rate, especially with the acceleration of food prices.

Second, pregnancy increases the vulnerability to quitting one’s job. The quantitative survey found that 7.1% of the participants quit their jobs due to pregnancy, while qualitative exploration revealed that participants left their jobs most frequently during the first trimester, with hyperemesis gravidarum (HG) being the most cited pregnancy-related cause. This finding contradicts that of Laraia [74], who reported that pregnant women typically stopped working in the third trimester. Meanwhile, for multipara, a lack of childcare typically forces them to quit their jobs following the birth of a previous child. Nonetheless, the consequences of quitting jobs were the same: limiting household income and forcing a single earner (the husband) to support the family’s basic needs.

Thirdly, acute financial crises and lack of household savings resulted in severe food insecurity and hunger. In the quantitative phase, 20% of the participants were surveyed during Phase 1 of the MCO. The qualitative exploration revealed that the COVID-19 pandemic led to sudden retrenchments, wage reductions, and unpaid leaves. The situation became dire when the family’s sole breadwinner was affected, especially when they had no household savings. This aligns with a World Bank [73] report indicating that most Malaysian households lack savings and that most working individuals struggle financially, regardless of location or age group. Additionally, according to Ganeshwaran Kana [75], the COVID-19 pandemic shifted over 60,000 (about 8%) Malaysian M40 households fall into the B40 income group. Therefore, when food accessibility and availability were limited, the portion size and frequency of food consumption were reduced [76], either to ensure food for the following day or to ensure sufficient food for children by compromising own’s nutritional needs among pregnant women as part of coping mechanism, particularly among multipara. Conversely, those with secure employment or family support, notably working or retired parents, were spared from experiencing or exacerbating pre-existing food insecurity during the COVID-19 pandemic.

Studies have shown that food insecurity triggers chronic stress [74], which activates the hypothalamic–pituitary–adrenal (HPA) axis and leads to elevated cortisol levels. Prolonged exposure to cortisol can disrupt maternal weight regulation, potentially causing both excessive and inadequate gestational weight gain (GWG). Cortisol also crosses the placenta, affecting feotal growth and development, which increases the risk of low birth weight, preterm birth, and developmental delays. Additionally, it may exacerbate pregnancy complications such as gestational diabetes (GDM) and pre-eclampsia. The combination of food insecurity, stress, poor nutrition, and hormonal imbalances heightens the risk of adverse pregnancy outcomes and long-term health complications for both the maternal and child [77].

The strength of this present study is that, to the best of our knowledge, it is the first population-based study to use a sequential explanatory mixed-methods approach to explore the factors influencing suboptimal GWG in Malaysia and to provide a pathway that highlights the effect of each predictor and its interplay with the surrounding environment. It combines the strengths of both quantitative and qualitative research while cross-validating the findings of each method, thus strengthening the validity of the results [21]. The use of SEM as an overarching framework, along with an in-depth exploration of different stages of women’s reproductive life, provided a more comprehensive understanding of both inadequate and excessive GWG, particularly in developing countries, and facilitated the development of targeted yet holistic interventions. Previous studies have only explored individual perspectives and were limited to excessive GWG outcomes. Interestingly, this study was able to capture the impact of the COVID-19 pandemic on GWG through the real-life experiences of the participants.

Nevertheless, this study is not without limitations. There was a significant time gap between the quantitative first phase and the qualitative second phase of this study due to the COVID-19 pandemic. Hence, there was a change in the sociocultural context, which could have resulted in recall bias. Nonetheless, every attempt was made to verify the timing of specific events. While no member checking was performed to ensure the validity of participant responses amidst the pandemic, this was minimized through methods and data triangulation. Additionally, given the objective of this study to identify and explain predictors of suboptimal GWG, there is a limitation in correlating participants’ perceptions with their health status through biochemical markers. Future studies should explore this area further. Lastly, as this was a qualitative study focusing on the experiences of pregnant women with GWG, only their perspectives were included. Thus, the study does not provide generalizable or direct information about the advice provided by HCPs, as the data were based on women’s self-reported behaviours and GWG-related experiences from a representative sample of a particular region. Future research should consider incorporating healthcare providers’ perspectives and examining effective interventions for suboptimal GWG that are tailored to the pregnant woman’s needs.

The findings of this study have significant policy implications for improving antenatal care services. This study highlights the need for enhanced pre-pregnancy care to address the rising prevalence of obesity among women of reproductive age [78], which increases the risk of suboptimal GWG and related complications [79]. Public health initiatives should focus on raising awareness about the risks of obesity and the importance of optimal GWG while addressing misconceptions such as the “eating for two” myth, the belief that exercise harms the foetus, and cravings, particularly through targeted social media campaigns. Healthcare providers (HCPs) should receive comprehensive training on maternal nutrition, offering early (i.e., pre-pregnancy or first trimester), culturally sensitive counseling on lifestyle, weight gain, exercise, and gestational diabetes prevention. Collaboration with dietitians will ensure personalized nutritional guidance based on pre-pregnancy BMI and individual social factors. Addressing challenges posed by pregnancy-related comorbidities, such as hyperemesis gravidarum and food taboos associated with specific health conditions, is crucial for supporting women in maintaining healthy eating habits. Additionally, given the critical role of lifestyle modifications in promoting lasting health outcomes, the involvement of experts, such as lifestyle coaches or maternal health counselors, is crucial in guiding women to make sustainable changes before, during, and after pregnancy. These experts can offer personalized support, assist in behaviour change, and provide education on maintaining healthy practices beyond the pregnancy period. Psychological screening and support, particularly at the time of diagnosis for conditions such as gestational diabetes [80], is crucial for ensuring proper nutrition and glycemic control, with timely referrals as needed. Close monitoring of GWG is recommended when nutrition is compromised (e.g., weight loss, poor weight gain, urine ketone positivity, and avoiding nutritious foods through dietary diaries). Finally, antenatal care should include screenings for food insecurity (e.g., the Hunger Vital Sign) [77], with multi-agency collaboration to provide timely food assistance or subsidiary programs.

## 5. Conclusions

In conclusion, suboptimal gestational weight gain (GWG) resulted from the complex interplay of intrapersonal, interpersonal, social, and organizational factors, as well as local and national policies. This study demonstrates how pre-pregnancy overweight or obesity, diabetes during pregnancy, and low- to middle-income households contribute to suboptimal GWG through interactions between personal, social, and healthcare-related factors. Quantitative findings have identified significant associations, while qualitative insights clarified extended phenomena such as unhealthy lifestyle persistence, emotional responses to diagnoses, financial constraints, and healthcare access. By integrating both methods, this mixed-method study provides a comprehensive view of the factors influencing suboptimal GWG, relevant not only to Malaysia but also to other developing countries. These findings highlight the need for targeted interventions that address the multifaceted perspectives of suboptimal GWG.

## Data Availability

The data are available upon request to the corresponding author. The data are not publicly available due to privacy restrictions.

## Supplementary Materials

The following supporting information can be downloaded at: https://www.mdpi.com/article/10.3390/healthcare13101099/s1, Figure S1: Study flowchart; Table S1: Summary of sociodemographic and obstetric characteristics of the respondents interviewed. Table S2: Themes, subthemes and categories of inadequate and excessive gestational weight gain in Selangor. Method S1: Interview guide.

## Abbreviations

The following abbreviations are used in this manuscript:

BMI	Body Mass Index
GDM	Gestational Diabetes Mellitus
GWG	Gestational weight gain
HCP	Healthcare provider
MCO	Movement control order
