# Supplementary material for: From “Eating for Two” to Food Insecurity: Understanding Weight Gain Perspective During Pregnancy Among Malaysian Women
Source: Healthcare (Basel). 2025 May 8;13(10):1099. doi: 10.3390/healthcare13101099 (PMC12111471; doi:10.3390/healthcare13101099)
Supplement: Supplementary file 1 [file healthcare-13-01099-s001.zip › healthcare-3570446-supplementary/Method S1.pdf]

Method S1.

Interview Guide

1. What do you understand about pregnant weight issues?
2. Could you tell me what factors contributed to your pre-pregnancy overweight/obesity? What affect does it have on your weight?
3. Could you please describe your experience with weight changes during pregnancy? Could you elaborate?
4. Could you tell me what contributed to your weight changes during pregnancy? Could you explain more?
5. Could you tell me how your diet has changed since you became pregnant?
6. Could you describe how your physical activity changes during pregnancy in comparison to before?
7. Could you tell me about your experience of having diabetes during current pregnancy? Could you please elaborate?
8. How do you cope with diabetes?
9. Have you ever been concerned about food shortage or no food at home throughout your current pregnancy? Could you tell more about it?
