# Supplementary material for: From “Eating for Two” to Food Insecurity: Understanding Weight Gain Perspective During Pregnancy Among Malaysian Women
Source: Healthcare (Basel). 2025 May 8;13(10):1099. doi: 10.3390/healthcare13101099 (PMC12111471; doi:10.3390/healthcare13101099)
Supplement: Supplementary file 1 [file healthcare-13-01099-s001.zip › healthcare-3570446-supplementary/Table S1.pdf]

**Table S1.** Summary of sociodemographic and obstetric characteristics of the respondents interviewed (n=20)

| ID | Age | Parity | Education level | Diabetes in pregnancy status | Employment status | Gestational age (weeks) | Pre-pregnancy BMI | GWG status |
|---|---|---|---|---|---|---|---|---|
| R1 | 33 | G5P4 | Secondary | GDM ( diet) | Housewife/ stop working due to pregnancy | 37 | Obese | Excessive |
| R2 | 28 | 1 | Pre-university | Non-diabetic | Chartering executive | Postpartum (4 months) | Overweight | Excessive |
| R3 | 35 | 1 | Secondary | GDM (diet) | Dispensing operator | Postpartum (2 months) | Obese | Excessive |
| R4 | 26 | G1P0 | Pre-university | GDM | Account clerk | 34 weeks | Obese | Inadequate |
| R5 | 28 | 1 | Tertiary | Non-diabetic | Teacher | Postpartum (3 months) | Obese | Excessive |
| R6 | 31 | 1 | Secondary | Non-diabetic | Housewife | Postpartum (2 months) | Overweight | Excessive |
| R7 | 31 | 4 | Pre-university | GDM (Metformin) | Executive | Postpartum ( 2 months) | Obese | Inadequate |
| R8 | 37 | 4 | Tertiary | GDM (diet) | Housewife/ stop working due to pregnancy | Postpartum (5 months) | Obese | Inadequate |
| R9 | 21 | 2 | Secondary | GDM (diet) | Housewife/ stop working due to pregnancy | Postpartum (2 months) | Obese | Inadequate |
| R10 | 27 | 3 | Secondary | GDM (MTF) | Housewife | Postpartum (3 months) | Obese | Inadequate |
| R11 | 31 | 2 | Tertiary | GDM (diet) | Housewife | Postpartum (6 months) | Obese | Inadequate |
| R12 | 28 | 1 | Tertiary | Non-diabetic | Optometrist | Postpartum (5 months) | Underweight | Inadequate |
| R13 | 25 | 1 | Tertiary | Non-diabetic | Housewife | Postpartum (7 months) | Overweight | Inadequate |
| R14 | 29 | 1 | Tertiary | Non-diabetic | School canteen cashier | Postpartum (4 months) | Obese | Inadequate |
| R15 | 28 | 4 | Secondary | GDM (insulin) | Admin | Postpartum (6 months) | Overweight | Inadequate |
| R16 | 20 | 1 | Pre-university | Non-diabetic | Housewife | Postpartum (7 months) | Obese | Inadequate |
| R17 | 25 | 2 | Pre-university | Non-diabetic | Housewife/ stop working due to pregnancy | Postpartum (6 months) | Obese | Inadequate |
| R18 | 26 | 2 | Pre-university | Non-diabetic | Accountant assistant | Postpartum (5 months) | Obese | Inadequate |
| R19 | 32 | 3 | Secondary | Non-diabetic | Admin | Postpartum (4 months) | Obese | Inadequate |
| R20 | 24 | 2 | Secondary | Non-diabetic | Housewife | Postpartum (2 months) | Overweight | Excessive |
