# Supplementary material for: From “Eating for Two” to Food Insecurity: Understanding Weight Gain Perspective During Pregnancy Among Malaysian Women
Source: Healthcare (Basel). 2025 May 8;13(10):1099. doi: 10.3390/healthcare13101099 (PMC12111471; doi:10.3390/healthcare13101099)
Supplement: Supplementary file 1 [file healthcare-13-01099-s001.zip › healthcare-3570446-supplementary/Table S2.pdf]

Table S1. Themes, subthemes and categories of inadequate and excessive gestational weight gain in Selangor.

Outcome	Theme	Sub-themes	Categories	Quote
Excessive GWG	1. The impact of pre-pregnancy overweight and obesity	Unhealthy pre-pregnancy lifestyle	Unhealthy pre-pregnancy diet preferences.	Before pregnancy, I ate little rice but a lot of junk food like chips, snacks, and pickles. (R2, para 1)
			Work nature and built environment promoting unhealthy diets and sedentary lifestyles.	When I'm bored (waiting customer), I eat (chips). (R3, para 1)
			Limited disposable income forced the purchase of cheap but low-quality food.	We had little money then, so my roommate and I relied on instant noodles. (R6, para 1)
		Pregnancy lifestyle changes	Continuation of pre-pregnancy unhealthy eating habits and sedentary lifestyle.	I eat exactly like when I wasn't pregnant. I'll eat whatever I want. (R15, para 4)
			Unhealthy pregnancy-related dietary behaviour changes	I wasn't a fan of sweets, but now I'm pregnant, I crave them, especially cakes. I can eat two slices at a time. (R5, para 1)
			Pregnancy-related physical discomfort	Tired. My body feels heavy. I get out of breath when walking. (R20, para 2)
			Pregnancy overeating prior to childbirth and confinement	Later during confinement, I won't be able to eat, so before giving birth, I eat a lot. (R6, para 1)
			MCO-related unhealthy lifestyle	Yes, I ate a lot during the MCO. Everyone did. Staying at home was boring [chuckle]. I ate constantly and quickly ran out of money without realizing it. (R8, para 4)

Table S1. Continued

Outcome	Theme	Sub-themes	Categories	Quote
Excessive GWG	The impact of pre-pregnancy overweight and obesity (Socioecological model)	Pregnancy related beliefs	Overeating and gaining weight is normal during pregnancy.	Everyone gains weight during pregnancy; it's normal (R3, para1)
			Pregnancy is seen as a time to rest and enjoy foods usually avoided.	Before pregnancy, I had to diet and couldn't eat this or that. Now that I'm pregnant, I can eat everything—it feels like heaven. (R5, para1)
			Craving, uncontrollable appetite, aversion, hunger, lethargy are perceived as the baby's way of communicating.<br>Eating for two	Whatever we feel like eating, it's not from us, it's the baby. The baby wants to eat it. (R5, para 1)
			Concern about providing adequate nutrition for the baby and not depriving them.	Now, I am pregnant, I have to eat for two. (R12, para 1)
			Maternal weight is not an issue as long as the baby is healthy.	The baby is hungry [...] Pity the baby. So, I have to wake up in the middle of the night to eat. (R9, para2)
			Fear that physical activity may harm the baby.	I don't mind my weight; what matters is the baby. (R2, para1)
				I'm scared that I might harm the baby (R14, para1)

Table S1. Continued

Outcome	Theme	Sub-themes	Categories	Quote
Excessive GWG	The impact of pre-pregnancy overweight and obesity	Social network pressure	Pressuring mothers to eat.	My father in law said, "You can eat at 8pm, but you must eat again after Isha prayers (8.30pm). If not, the baby will be hungry. Don't restrict eating, it's not good." (R5, para 1)
			Preventing physical activities.	I didn't do any housework during my pregnancy. My husband did everything; cooking, laundry. (R 12, para1)
			Family and friends have unhealthy eating habits.	My husband's family all love eating late at night, so I have to follow them (R16, para 1)
			Similar eating behaviours and accompanying buddies	We all (colleagues) love eating desserts. We order lots of food, eat together, and finish everything. (R3, para 1)
				Because I didn't have anyone to accompany me (for exercise). My husband works the morning shift from Monday to Sunday with no days off (R14, para 1)
			Social network consider weight management is only important before pregnancy	Before, my husband insisted I diet, exercise, and take care of my body. Now that I'm pregnant, he keeps telling me to eat and eat.
			Judgement on weight and body image	My friend said, "It's okay to eat a lot, after giving birth, you'll lose the weight."

Table S1. Continued

Outcome	Theme	Sub-themes	Categories	Quote
Excessive GWG	The impact of pre-pregnancy overweight and obesity	Health care providers (HCP) interactions	Reliance on healthcare providers to alert mothers about weight issues, along with inconsistent and vague advice	Since I wasn't referred to a doctor, I believed I was still well. If it was urgent and dangerous, I would have been referred to a doctor, right? (R5,para 1)
			Busy clinics hampering healthcare provider interactions.	I couldn't ask because the doctor was busy.(R5, para 1)
			Insensitive comments about GWG	I assumed no matter how much weight I gained or lost, I would be admonished. So, I just didn't bother [laughs] (R20, para 2)
		Limited accessibility	Unaffordable healthy foods and prenatal exercise courses.	The prenatal class is expensive, I can't afford it.
			Fluctuating food supplies.	I asked my husband how much money he had today, and he said RM10. When I asked him what we were going to eat, he said, "We just eat burgers."(R2, para 1)
		No comorbidities	Continuation of an unhealthy lifestyle during pregnancy leading to excessive GWG	Yes, I eat all the time. I think that's the reason for my weight gain. Some people experience morning sickness and can't eat, but I didn't have morning sickness. When I went for check-ups, everything was fine—I didn't have high blood pressure, diabetes, or any other issues (R6, para 1)

Table S1. Continued

Outcome	Theme	Sub themes	Categories	Quote
Inadequate GWG	2.Managing diabetes during pregnancy	Psychological responses to diabetes diagnosis	The diagnosis of diabetes during pregnancy serves as a wake-up call.<br><br>The emotional impact and fear.	I was diagnosed with GDM. Since then, I have controlled my food intake. (R20, para 1)<br><br>I was devastated when I found out I had diabetes. (R4, para 1)
		Overcompensation and restrictive behaviours	Dietary changes and a shift toward extreme dietary restriction.	I fasted a lot while pregnant. I didn't eat much during my fasts, just Jacob's milk crackers the whole day. I lost weight. (R9, para 2).<br><br>The doctor told me to eat rice twice a day, but I only ate it once because I was afraid my blood sugar profile (BSP) would rise. (R17, para 2)
		Prioritization of fetal health	Health of baby prioritized over maternal nutritional needs.	I am not afraid of the weight. I am more worried of the sugar control and my baby (R8, para4)
		Barriers to accessing timely and comprehensive dietary guidance	HCP prioritised diabetes control over nutritional adequacy<br><br>Long waiting period for a dietitian's appointment.	My weight? Not really. They mainly focused on my diabetes. (R7, para 4)<br><br>I waited almost a month to see one. At that time, my BGLs were high and uncontrolled. [..] I ate less and started losing weight. (R4, para 1)

Table S1. Continued

Outcome	Theme	Sub themes	Categories	Quote
Inadequate GWG	3.The influence of middle and low household income	Income vulnerability in pregnancy	Financial hardship and multiple obligations.	My income is fully used for commitments. At the end of the day, I have only RM200 or RM300 left to save for the baby. (R2, M40)
			Pregnancy-related job instability, quitting work due to pregnancy, and constrained household finance	I finally gave up and resigned from my job. My morning sickness made it difficult to work. (R15, B40)
		Financial crisis and food insecurity	No saving and acute financial crises leading to food insecurity and hunger.	My husband usually gets paid monthly, but since the MCO began, he gets paid daily. He's only called if there's work. He hasn't been hired in a month, so it's affecting our income. (R17, B40)<br>We eat whatever is available. One tin of sardines, I divide into four meals for my child. My husband and I don't eat. (R10, B40)
