# Supplementary figures and images for: From “Eating for Two” to Food Insecurity: Understanding Weight Gain Perspective During Pregnancy Among Malaysian Women
Source: Healthcare (Basel). 2025 May 8;13(10):1099. doi: 10.3390/healthcare13101099 (PMC12111471; doi:10.3390/healthcare13101099)

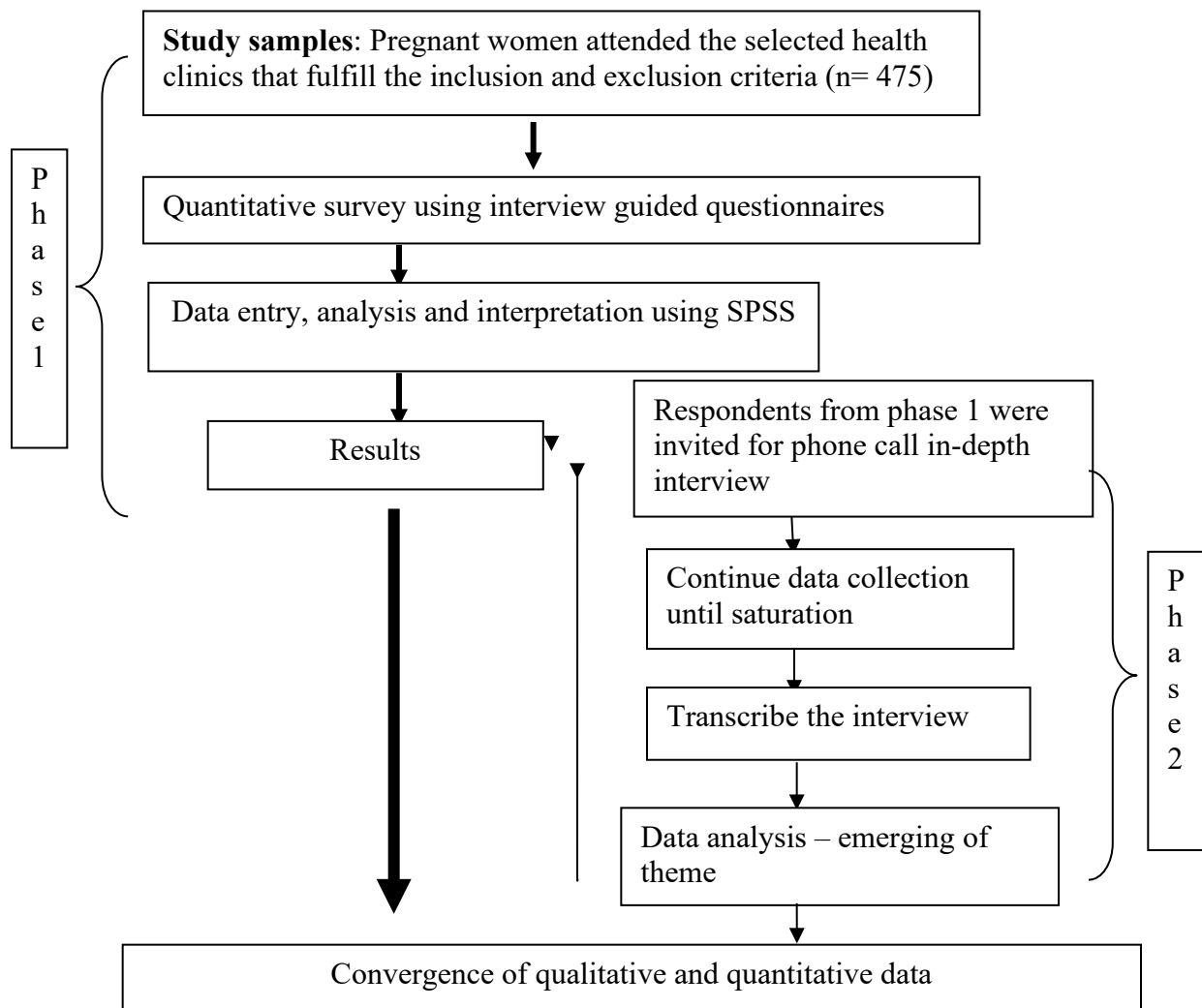

**Figure S1:** Study flowchart

Supplement: Supplementary file 1 [file healthcare-13-01099-s001.zip › healthcare-3570446-supplementary/Figure S1.pdf]
